# Biochemical and Physiological Responses of Weeds to the Application of a Botanical Herbicide Based on Cinnamon Essential Oil

**DOI:** 10.3390/plants13233432

**Published:** 2024-12-06

**Authors:** Sofiene Ben Kaab, Juan Antonio Fernández Pierna, Berenice Foncoux, Philippe Compère, Vincent Baeten, M. Haïssam Jijakli

**Affiliations:** 1 Integrated and Urban Plant Pathology Laboratory, University of Liège, Gembloux Agro-Bio Tech, 2 Passage des Déportés, 5030 Gembloux, Belgium; 2 Quality and Authentication of Products Unit, Knowledge and Valorization of Agricultural Products Department, Walloon Agricultural Research Centre (CRA-W), Chaussée de Namur 24, 5030 Gembloux, Belgium; 3 Laboratory of Functional Morphology and Evolution, Center for Applied Research and Education in Microscopy (CAREM), University of Liège, 4000 Liège, Belgium; 4 Biomaterials Interfaculty Center (CEIB), University of Liège, 4000 Liège, Belgium

**Keywords:** phytotoxic effect, cinnamon essential oil, transmission electron microscopy (TEM), membrane integrity and permeability, weeds, malondialdehyde (MDA)

## Abstract

The use of chemical herbicides induces negative impacts on the environment, animals, and human health. It also leads to the development of herbicide-resistant weeds. In this context, natural and efficacious herbicides are highly sought after. Essential oils are natural compounds with antibacterial, fungicidal, and phytotoxic properties. For this reason, we studied the post-emergence phytotoxic effect of cinnamon essential oil (cinnamon EO) from *Cinnamomum cassia* under greenhouse conditions, testing it against *Trifolium incarnatum* (*T. incarnatum*) and *Lolium perenne* (*L. perenne*). The content of malondialdehyde (MDA), percentage of water loss, electrolyte leakage, and the fluorescence of treated leaves by cinnamon EO were determined in order to understand the physiological and biochemical responses. In addition, transmission electron microscopy (TEM) was used to study the effect of cinnamon EO on cellular organelles in different tissues of *T. incarnatum* leaves. Results showed that cinnamon EO quickly induced oxidative stress in treated leaves by increasing MDA content, impacting membrane integrity and causing water loss. TEM observations confirmed the cell desiccation by cellular plasmolysis and showed an alteration of the membrane integrity and chloroplast damages. Moreover, Raman analysis confirms the disturbance of the plant metabolism by the disappearance of some scattering bands which correspond to primary metabolites. Through our finding, we confirm that cinnamon essential oil (EO) could be proposed in the future as a potential bioherbicide and a suitable source of natural phytotoxic compounds with a multisite action on weeds.

## 1. Introduction

Crop losses due to weeds continue to reduce available production worldwide. These yield losses (about 32%) are greater than those caused by pests (18%) or pathogens (15%) [1]. They can cause dramatic changes in ecological systems and agricultural fields, as they profoundly alter communities and ecosystems [2]. Economic losses are estimated to be 30% to 93% in maize [3], 12% to 61% in potato [4], and 7 to 25% in wheat [5]. Moreover, weeds can pose health risks by contaminating food and feed products such as in the case of *Datura stramonium*, which is toxic and attacks various organs including the liver, heart, kidneys, and brain. For these reasons, weed management will be of crucial importance to ensure global food security and safety [6].

The use of chemical herbicides is the most common way to manage weeds in the field. However, the excessive use of these synthetic herbicides has been shown to induce adverse impacts on the environment, animals, and human health due to their toxicity, persistence in the environment, and bioaccumulation in organisms [7]. For these reasons, the need to enhance crop production while preserving the ecological integrity of agroecosystems drives the search for innovative technologies, such as biopesticides, which offer promising alternatives to conventional methods [8]. Among these biopesticides, natural compounds, particularly essential oils (EOs), stand out as highly promising candidates. The latter have shown a high phytotoxic effect. The pre- and post-emergence efficacy of EOs against a variety of weeds has already been demonstrated in the literature [9,10,11,12,13,14]. Essential oils present less or no negative impact on human health and the environment. Their low risk for the environment can be explained by their rapid degradability in the environment, leaving no trace or residue in the soil or water [15]. Thus, they allow for a good ecological balance and the preservation of biodiversity. Moreover, it has been reported that EOs present a low toxicity for non-targeted organisms and a low risk for human health when used at usual concentrations or doses [16]. A few bioherbicides are already available in the EU market, such as Beloukha^®^, which contains pelargonic acid extracted from pelargonium oil, which is derived from the *Pelargonium* species, a plant in the *Geraniaceae* family [15].

Essential oils can be classified into two groups according to the biosynthetic origin of the dominant molecule(s), representing 20 to 70% of the extract; these groups are terpenoid essential oils and aromatic essential oils [17]. The terpenoids group constitutes the most diverse category of natural plant-based products. Their biosynthesis is related to the isopentenyl diphosphate precursor. They represent the largest class of natural products, with over 55,000 structurally diverse known compounds such as camphene, phellandrenes, menthol, geraniol, etc. [18]. The main terpenes are monoterpenes, which are formed from coupling two isoprene units (C_10_). On the other hand, aromatic EOs are derived from phenylpropanoid pathways; among the principal plant sources are cinnamon (*Cinnamomum cassia*), fennel (*Foeniculum vulgare*), and anise (*Pimpinella anisum*).

Traditionally, *Cinnamomum cassia*, commonly known as cassia or Chinese cinnamon, a species of evergreen tree in the Lauraceae family, has been used for its medicinal and pharmacological properties, particularly its antioxidant, neuroprotective, anticancer, and antidiabetic effects [19,20,21]. For example, it is known to treat gastritis and dyspepsia, blood circulation disturbances, and inflammatory diseases [22,23]. It has been showed by Choi et al., 2001 [24], that cinnamaldehyde, which is the main compound in cinnamon EO, also has several biological activities such as peripheral vasodilatory and antitumor activity. Cinnamon EO, like many EOs, has recently been studied for its pesticidal activity, mainly in laboratory settings [20,25,26]. Moreover, it is commercialized as an insecticide by Biocinn^®^ and a fungicide by Seican^®^ [27].

Moreover, the phytotoxic effect of EOs on plants has been widely reported for the last 20 years. Verdeguer et al., 2020 [28], summarized the phytotoxic effect of several EOs. Most of them have been tested in seed germination and seedling growth. Overall, the determination of the mechanism of actions of EO toxicity was carried out only with classical analytical tools which consisted of measuring chlorophyll content, MDA, reactive oxygen species (ROS), and antioxidant enzymes. However, these findings were not confirmed using cutting-edge techniques such as omics methods, high-resolution imaging methods, the Raman spectroscopy method, etc. Interestingly, the latter method was used for the first time to study the effect of EOs on plant metabolism [29]. It is a highly appropriate technique for this task. However, this technology has several applications across diverse fields, with its notable contribution to chemical biology standing out prominently. It is used in the agriculture field to control and monitor the quality of feed by providing many details about molecular vibrations and facilitates the detection of potential alterations in cell structure. Its sensitivity to small structural changes, non-invasive sampling capacity, minimal sample preparation, and high spatial resolution are part of its advantages [30,31].

Regarding the phytotoxic effect of cinnamon EO, it was initially documented by Tworkoski, 2002 [32], and recently by Werrie et al., 2022 [33]. The latter group of authors explored the potential phytotoxicity of cinnamon EO on apple trees (*Malus domestica*). They discovered that it induced oxidative damage by generating a high level of malondialdehyde using a molecular approach. On the other hand, Lins et al., 2019 [34], also investigated the phytotoxic effect of cinnamaldehyde by characterizing its interaction with the plant plasma membrane using an integrative biophysical approach. In brief, all these findings concerning the phytotoxic effect of cinnamon EO did not include solid phenotypic experiments with dose–response data against both dicotyledonous and monocotyledonous species or microscopic images of treated leaves showing the ultrastructural changes caused by EO. These experiments should be performed to support their results, as abiotic stress during experiments can disrupt membrane integrity, decrease photosynthetic performance, and induce oxidative stress, leading to increased MDA content and ROS production. For these reasons, our study aims to evaluate the phytotoxic effect of cinnamon EO using various tools, including transmission electron microscopy (TEM) and Raman spectroscopy, to confirm its effectiveness against both dicotyledonous and monocotyledonous species. The combination of classical analytical tools with the TEM technique and Raman spectroscopy method was never applied before to such an EO. This approach enables the correlation of phenotypic changes at the plant level with physiological and biochemical changes at the cellular level, unveiling the potential of CEO as a botanical bioherbicide. Additionally, it narrows the search for the molecular mechanisms of action that could explain how cinnamon EO causes injury in plants. To do so, we study the phytotoxic effect of cinnamon EO against *Trifolium incarnatum* (*T. incarnatum*) and *Lolium perenne* (*L. perenne*) and evaluated the photosynthesis activity and membrane integrity of plant leaves using a fluorimeter and conductivity meter, respectively. The MDA content was also measured by optic spectroscopy to study the effect of EO on the plant metabolism, especially in terms of oxidative stress. Moreover, the ultrastructure of leaf cells of different tissues of *T. incanatum* treated by different concentrations of EO was observed using TEM to confirm results and understand the main cellular damages. Finally, we also used Raman spectroscopy to detect the potential alterations in metabolism of *T. incarnatum*.

## 2. Results

### 2.1. Phytotoxic Effect of Cinnamon EO on T. incarnatum and L. perenne 

**Figure 1:**
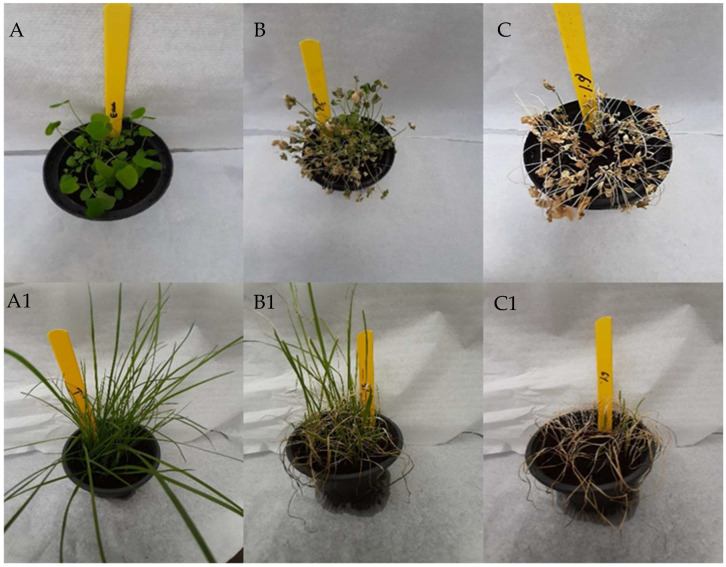
Phytotoxic effect of cinnamon EO after three days on *T. incarnatum* (**upper**) and *L. perenne* (**lower**). From left to right: untreated ((**A**) or (**A1**)); treated plant with 3% EO ((**B**) or (**B1**)); treated plant with 6% EO ((**C**) or (**C1**)).

To investigate the effect of cinnamon essential oil (EO) on different weed species, we tested the monocotyledonous weed *L. perenne* and the dicotyledonous weed *T. incarnatum*. The results showed that cinnamon EO exhibits a strong phytotoxicity effect against both weed species in a concentration-dependent manner. Specifically, the phototoxicity was most pronounced in plants treated with 6% cinnamon EO, as evidenced by significant wilting, necrosis, and chlorosis of the leaves observed three days after spraying (Figure 1).

### 2.2. Effect of Cinnamon EO on Physiological and Biochemical Parameters of Weeds

Due to the quick desiccation effect caused by spraying cinnamon EO on weed plants, we assessed several physiological and biochemical parameters using classical analytical tools.

**Figure 2:**
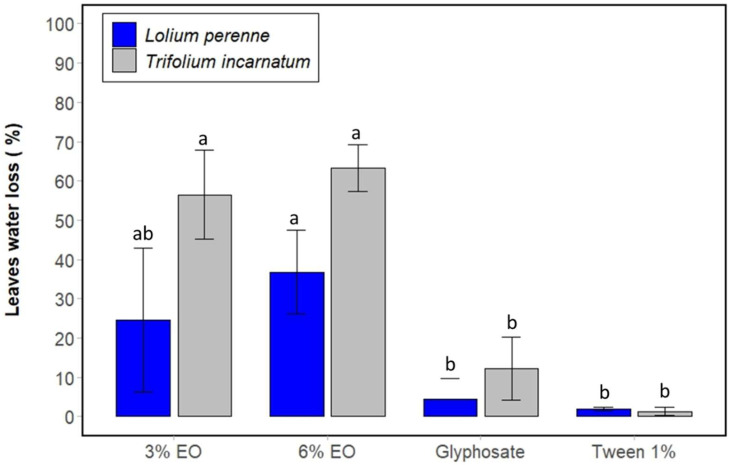
Percentage of water loss of leaves of *T. incarnatum* (gray) and *L. perenne* (blue) treated by cinnamon EO at 3% and 6% after 5 h. Glyphosate at 7 g L^−1^ was used as positive control and Tween 20 (1%) as negative control for emulsion of cinnamon EO. ^a,b^ The letters above the histogram bars represent statistical groups. Values in a column followed by the same letter are not significantly different at *p* < 0.05, as established by Tukey’s test, indicating that these groups do not differ statistically from one another.

**Figure 3:**
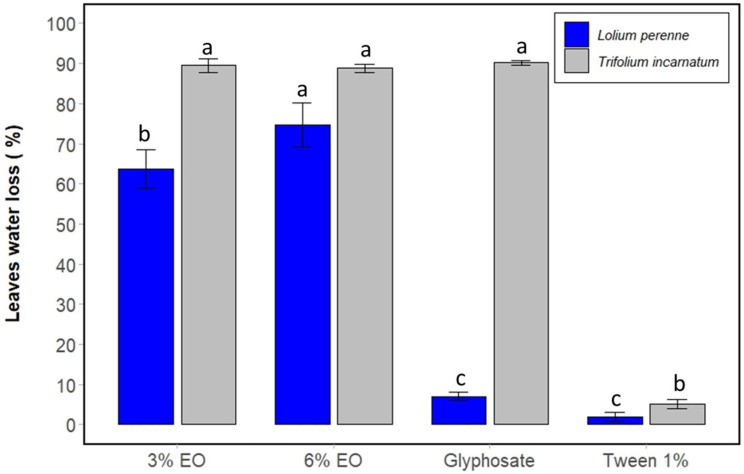
Percentage of water loss of leaves of *T. incarnatum* (gray) and *L. perenne* (blue) treated by cinnamon EO at 3% and 6% after 3 days. Glyphosate at 7 g L^−1^ was used as positive control and Tween 20 (1%) as negative control for emulsion of cinnamon EO. ^a–c^ The letters above the histogram bars represent statistical groups. Values in a column followed by the same letter are not significantly different at *p* < 0.05, as established by Tukey’s test, indicating that these groups do not differ statistically from one another.

First of all, the desiccant effect was evaluated by calculating the water loss in the leaves resulting from the application of cinnamon EO. In fact, statistical analysis tests showed that cinnamon EO significantly decreases the water content of the leaves of *T. incarnatum* and *L. perenne* compared to the control. At 3% and 6%, the essential oil decreases the water content by approximately 56% and 63% for *T. incarnatum* and 24% and 36% for *L. perenne* compared to the control. This effect was accentuated after three days, with water loss reaching 88% and 89% for *T. incarnatum* leaves compared with 63% and 74% for *L. perenne*. It is also clear that *L. perenne* shows fewer symptoms of desiccation than *T. incarnatum* (Figure 2 and Figure 3).

**Figure 4:**
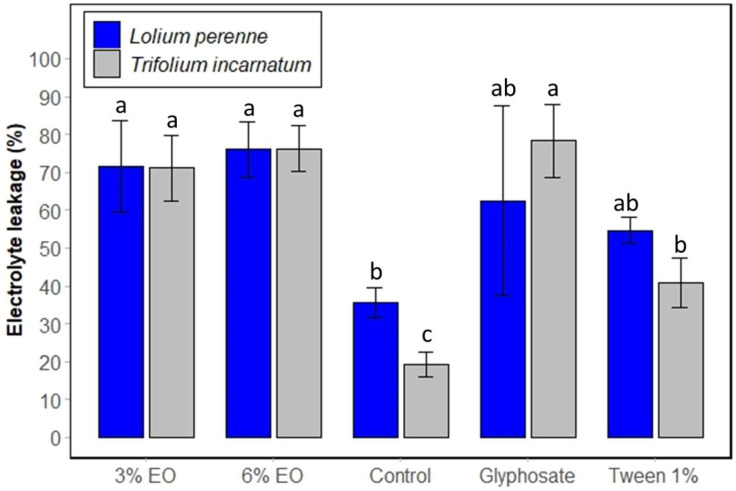
Membrane integrity of *T. incarnatum* (gray) and *L. perenne* (blue) after 5 h of treatment with cinnamon EO 3% and 6%. Glyphosate at 7 g L^−1^ was used as positive control and Tween 20 (1%) as negative control for emulsion of cinnamon EO. ^a–c^ The letters above the histogram bars represent statistical groups. Values in a column followed by the same letter are not significantly different at *p* < 0.05, as established by Tukey’s test, indicating that these groups do not differ statistically from one another.

Secondly, the relative electrolyte leakage (REL) parameter was evaluated to estimate the membrane integrity in the plant leaves treated by cinnamon EO. A higher REL percentage compared to the control signifies damage to the cell membrane. That is what Figure 4 shows. In fact, cinnamon EO exhibited high electrolyte leakage levels in both plant species, reaching 76.30% for *T. incarnatum* and 75.98% for *L. perenne*. These values are significantly higher than those of the control, which were 19.31% for *T. incarnatum* and 35.69% for *L. perenne.* The statistical analysis confirms this high significant difference in terms of electrolyte leakage between untreated leaves and leaves treated with cinnamon EO at 3%, cinnamon EO at 6%, and glyphosate. Concerning the glyphosate treatment, these percentages are slightly lower than those for *T. incarnatum* (89.35%) and slightly higher for *L. perenne* (62.56%).

**Figure 5:**
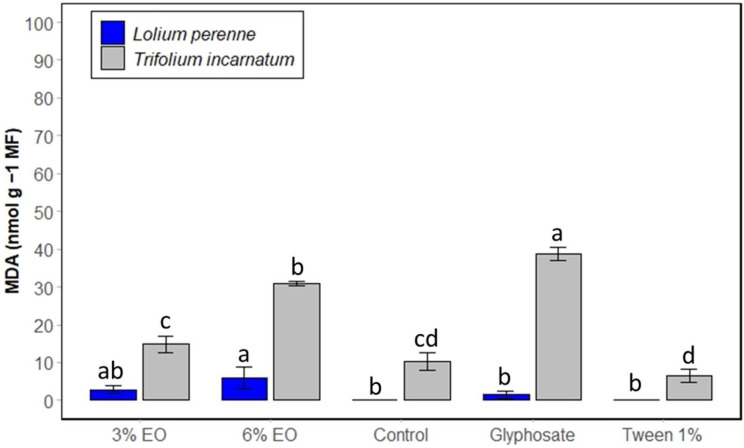
Content of MDA In *T. incarnatum* (gray) and *L. perenne* (blue) after 5 h of treatment with cinnamon EO at 3% and 6%. Glyphosate at 7 g L^−1^ was used as positive control and Tween 20 (1%) as negative control. ^a–d^ The letters above the histogram bars represent statistical groups. Values in a column followed by the same letter are not significantly different at *p* < 0.05, as established by Tukey’s test, indicating that these groups do not differ statistically from one another.

Figure 5 shows that the MDA content in the treated leaves was affected by cinnamon EO treatment in a concentration-dependent manner, reaching 30.96 nmol g^−1^ MF for *T. incarnatum*, which is three times higher compared to the control. The MDA content in *T. incarnatum* leaves treated with glyphosate was significantly higher than in other treatments, reaching 38.70 nmol g^−1^ MF. Regarding the MDA content in *L. perenne*, the effect of cinnamon EO was less pronounced, and even glyphosate had no significant impact.

**Figure 6:**
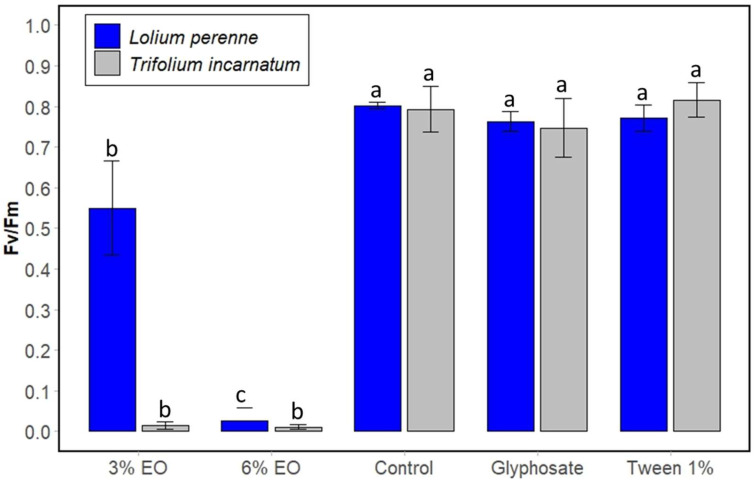
Chlorophyll fluorescence of leaves of *T. incarnatum* (gray) and *L. perenne* (blue) after 15 min of treatment with cinnamon EO 3% and 6%. Glyphosate at 7 g L^−1^ was used as positive control and Tween 20 (1%) as negative control for emulsion of cinnamon EO. ^a–c^ The letters above the histogram bars represent statistical groups. Values in a column followed by the same letter are not significantly different at *p* < 0.05, as established by Tukey’s test, indicating that these groups do not differ statistically from one another.

**Figure 7:**
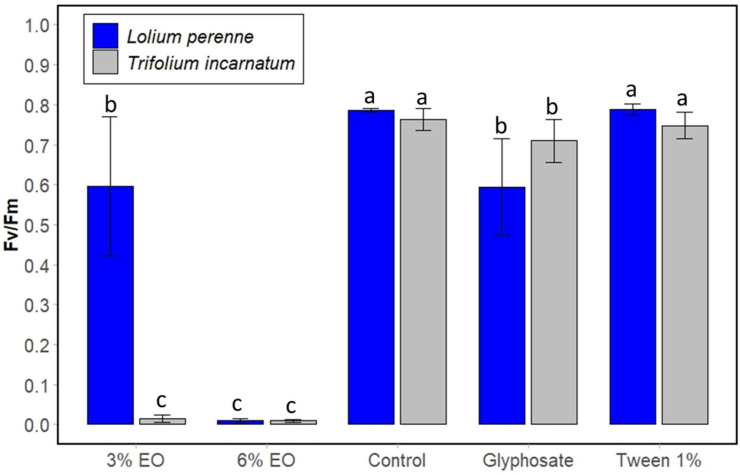
Chlorophyll fluorescence of leaves of *T. incarnatum* (gray) and *L. perenne* (blue) after 3 days of treatment with cinnamon EO 3% and 6%. Glyphosate at 7 g L^−1^ was used as positive control and Tween 20 (1%) as negative control for emulsion of cinnamon EO. ^a–c^ The letters above the histogram bars represent statistical groups. Values in a column followed by the same letter are not significantly different at *p* < 0.05, as established by Tukey’s test, indicating that these groups do not differ statistically from one another.

Finally, the chlorophyll fluorescence parameter was monitored to evaluate the photosynthetic performance of plant weeds. It is often used in several studies evaluating the phytotoxic effect of plant extracts. The maximum quantum yield of photosystem II primary photochemistry (Fv/Fm) was measured and compared between untreated and treated weed species at 15 min (Figure 6) and 3 days (Figure 7) following spraying. Figure 5 shows that cinnamon EO at 3% reduced the fluorescence of *T. incarnatum* and *L. perenne* leaves by 98.75% and 31.25%, respectively, compared to untreated leaves. At 6% of cinnamon EO, the chlorophyll fluorescence was completely inhibited for the two-plant species. Conversely, the application of Tween 20 (1%) showed no significant effect on fluorescence after 15 min and 3 days, reaffirming its non-phytotoxic effect. Concerning the glyphosate, it showed no effect on the fluorescence of plant leaves after 15 min of treatment but acted slightly after 3 days. The statistical analysis confirms that cinnamon EO significantly decreased the fluorescence of the plant leaves.

### 2.3. Effect of Cinnamon EO on Leaf Ultrastructure Determined by Transmission Electron Microscopy (TEM)

**Figure 8:**
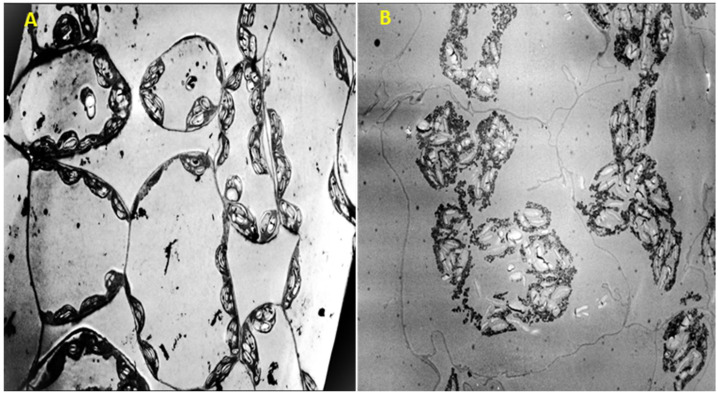
TEM images (600 × 200 µm) showing intact cells of untreated leaves of *T. incarnatum* containing Tween 1% (**A**) and the effect of cinnamon EO at 3% on the ultrastructure cells after 30 min of contact (**B**).

At the ultrastructural level, TEM images of parenchyma cells revealed significant changes and severe damages caused by cinnamon EO at 3% during the first 30 min of treatment. These observations clearly demonstrate the loss of the vacuole and the detachment of the cytoplasm from the cell wall (Figure 8B) in contrast to the control, which showed a large vacuole that takes up most of the cell’s area (Figure 8A). Additionally, TEM images of treated leaves indicate that chloroplasts and the large starch grains in the cytoplasm moved toward the center of the cells (Figure 8B), whereas the control cells maintained intact plasma membranes and cytoplasm adhering well to the cell walls, which keeps these organelles well inside (Figure 8A).

### 2.4. Effect of Cinnamon EO on Plant Metabolism of T. incarnatum Using Raman Spectroscopy

**Figure 9:**
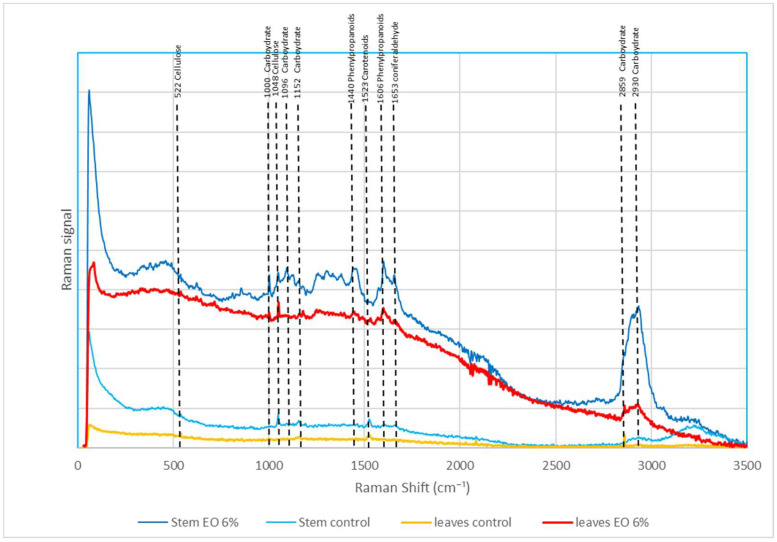
Raman spectra profile of untreated leaves (yellow) and stems (light blue) and treated leaves (red) and stems (dark blue) of *T. incarnatum* by cinnamon EO after 1 day.

The aim of this test was to compare the spectral profile in untreated *T. incarnatum* (leaves, stem, and roots) with treated *T. incarnatum* by cinnamon EO and consequently study the metabolic activity (Table 1). We noticed that the tissues treated with cinnamon EO show different spectral shapes compared to the control due to the appearance of two additional scattering bands (1440, 1606) and at the same time, the disappearance of some scattering bands (1152, 1523, 2859) (Figure 9). Roots were also tested, and the Raman signal was very weak.

## 3. Materials and Methods

### 3.1. Preparation of Herbicide Emulsion Based on Cinnamon EO 

Cinnamon EO was purchased from Vossen & co (Av. Van Volxem 264/C1, Bruxelles, Belgium). The technical data sheet was determined by GC-MS and showed that the main component is trans-cinnamaldehyde at 75%. Three and six milliliters of cinnamon EO were each mixed with 1 mL of Tween 20, which was used as an emulsifier. The final volume of each mixture was brought up to 100 mL with distilled water. The final solutions were immediately vortexed for 5 min to ensure the essential oil was well homogenized. It remained stable throughout the testing period. In fact, Tween 20 contains amphiphilic substances that facilitate interactions between polar and non-polar components. It was also applied to the plants that served as a control. Moreover, the concentrations of the EO were selected based on preliminary tests and the literature references [34,37].

### 3.2. Evaluation of Post-Emergence Activity of Cinnamon EO Under Greenhouse Conditions

Seeds of *T. incarnatum* and *L. perenne*, obtained from ECOSEM in Belgium, were sown in pots with a diameter of 11 cm, each filled with a standard potting mix (Universel, La Plaine Chassart, Fleurus, Belgium). *T. incarnatum* was selected because it is very common in the resident flora and has a very rapid growth in the greenhouse, while *L. perenne* was chosen due to its resistance to chemical herbicides. The plants were watered daily to maintain adequate soil moisture and promote uniform germination and growth. The greenhouse was maintained at a natural photoperiod supplemented with artificial light if needed, with temperatures set at 20 ± 3 °C according to sunlight. The relative humidity was maintained at 60 ± 3%.

Once the plants reached the 2–3-leaf stage, the five following solutions were sprayed on leaves using a small trigger sprayer (100 mL): (1) negative control with only water; (2) negative control containing 1% Tween 20; (3) cinnamon EO at 3% to evaluate its phytotoxic effect at a lower concentration; (4) cinnamon EO at 6% to evaluate its efficacy at a higher concentration; and (5) positive control containing glyphosate at 7 g/L, a commercial herbicide (Roundup^®^, 360 g a.i. L^−1^, Monsanto, St. Louis, MO, USA). Five replicates were conducted for each treatment in a completely randomized design. The treated plants were examined 5 h and 3 days after spraying to assess symptoms such as wilting, necrosis, and chlorosis. Subsequently, the treated leaves were harvested using scissors, placed in tubes, and stored at 4 °C. These leaves were then examined to assess several physiological and biochemical parameters, including water loss, chlorophyll fluorescence, malondialdehyde (MDA) content, and relative electrolyte leakage (REL).

### 3.3. Evaluation of Biochemical Parameters of Treated Weeds with Cinnamon EO 

#### 3.3.1. Water Loss 

The leaf samples collected from the post-emergence test were immediately weighed and placed in an oven at 70 °C for 3 days, then weighted again. The percentage of water in the leaves is calculated by Equation (1).

(1) $$Percentage\;of\;water\;in\;leaves\;\left(\%\right)=\frac{\;fresh\;weight-dry\;weight}{fresh\;weight}*100$$

#### 3.3.2. Evaluation of Photosynthetic Performance by Chlorophyll Fluorescence

Fifteen minutes and one hour after spraying, the measurements of *T. incarnatum* and *L. perenne* leaf fluorescence was performed using a HandyPEA fluorimeter (Hansatech Instruments, Pentney, Norfolk, UK) to evaluate the performance of photosystem II (PSII). Plants were pre-adapted for 15 min in the dark before chlorophyll fluorescence measurements. The latter were taken in the central part of leaves using special clips and were performed in nine biological replicates for each treatment (three per plant). The maximum quantum yield of the photosystem II primary photochemistry (Fv/Fm) was used as a critical measure of photosynthetic efficacy.

#### 3.3.3. Evaluation of Lipid Peroxidation by Measurement of Malondialdehyde (MDA)

MDA is a final product of the lipid peroxidation of polyunsaturated fatty acids present in the cell membrane. That is why we measured its quantity in treated weed species to investigate the potential membrane degradation. MDA can also serve as an indicator of oxidative stress. It was measured according to the thiobarbituric acid (TBA) test using the protocol of Ben Kaab et al., 2020 [38], with some modifications. In fact, fresh leaf samples of each treatment were crushed in trichloroacetic acid (TCA) (10 mL, 0.1%, *w*/*v*) and centrifuged at 12,000× *g* for 15 min. Then, one milliliter of the supernatant was added to 4 mL of thiobarbituric acid (0.5%, *w*/*v*, in 20%, *w*/*v*, TCA). The mixtures were heated at 100 °C for 30 min, immediately transferred to an ice bath, and then centrifuged at 12,000× *g* for 15 min. The absorbance of the solutions was recorded at 532 nm and 600 nm. The presence of MDA in the treated leaves was indicated by a pink color. Its content was calculated using ε = 155 mM − 1 cm^−1^, expressed in nmol g^−1^ and calculated by Equation (2).

(2) $$MDA(nmol\;g-1)=\frac{\left(A532\;nm-A600\;nm\right)}{155}$$

#### 3.3.4. Evaluation of Membrane Integrity by Relative Electrolyte Leakage (REL) 

Electrolyte leakage was determined following the method described by Poonpaiboonpipat et al., 2013 [39], using a conductivity meter (Hach, Isnes, Belgium). In fact, fresh leaf samples (100 mg) of each treatment were incubated in a test tube containing 20 mL of distilled water, and the electro-conductivity of the solutions was recorded after 30 min (EC1). Thereafter, the test tubes containing leaf tissues were boiled at 100 °C for 15 min and conductivity of the same leaf samples was again measured (EC2). The relative electrolyte leakage (REL) is calculated using Equation (3).

(3) $$REL(\%)=\frac{EC1}{EC2}*100$$

EC: Electrolyte conductivity.

### 3.4. Microscopy Observations with TEM (Transmission Electron Microscope) 

Squares of 2 × 2 mm of each treated leaf were cut with a scalpel in the same place starting from the edge toward the central vein. The squares were then immersed in a fixative solution of 2.5% glutaraldehyde in 0.1 M sodium cacodylate buffer at pH 7.4 and rinsed three times in 0.2 M cacodylate buffer at pH 7.2. Treated leaves were prepared for transmission electron microscopy. They were postfixed in 1% osmium tetroxide (OsO_4_) in cacodylate buffer and rinsed 3 times for 10 min with MilliQ water. The samples were dehydrated through a graded ethanol series (30, 50, 70, 90, and 100%), then in propylene oxide before gradual impregnating and embedding in epoxy resin (AGAR 100) in a vacuum oven to remove remaining gasses and solvent. The leaf samples were oriented in flat silicone molds and the resin was left to polymerize for at least 3 days at 60 °C. For light and transmission electron microscopy, semithin (1–2 µm thick) and ultrathin (60–80 nm section) cuts were performed with a 45° diamond knife (Diatome) on an ultramicrotome Reichert Ultracut E. Semi-thin sections were coloured with Toluidine Blue 1% at pH 9.0. The ultrathin section was uranyl acetate (1% in 50% ethanol) and lead citrate (1% in water). The observation of semithin sections was carried out with a light microscope (CX21i, Olympus, Tokyo, Japan). Images were taken thanks to a Moticam 10+ (Motic, Hong Kong, China) linked to the software Motic Image Plus 3.0. The ultrathin sections were observed in the TEM/STEM Tecnai G2 Twin (FEI, Hillsboro, OR, USA) of the CAREM-ULiege (Cell for Applied Research and Education in Microscopy) working at an 80 kV accelerating voltage.

### 3.5. Evaluation of Plant Metabolism by Raman Measurements 

*T. incarnatum* leaves, stems, and roots were cut separately, ground in liquid nitrogen, and stored in Eppendorf tubes for Raman measurements. FT–Raman spectra were acquired on a Vertex 70—RAM II Bruker FT—Raman spectrometer (Bruker, Billerica, MA, USA). This instrument is equipped with a Nd:YAG laser (yttrium aluminum garnet crystal doped with triply ionized neodymium) with an output at 1064 nm (9398.5 cm^−1^). The maximum laser power is 1.5 W. The measurement accessory was pre-aligned, and only the *Z*-axis of the scattered light was adjusted to set the sample in the appropriate position regarding the local point. The RAM II spectrometer is equipped with a liquid nitrogen-cooled Ge detector. OPUS 8.2 software was used for the spectral acquisition. The laser power was set at 1500 mW, the resolution at 4 cm^−1^, and the number of scans at 128 for each spectrum. Each spectrum was then collected in 4 min intervals.

### 3.6. Statistical Analysis 

Statistical analysis was carried out with R software. The data were analyzed using R Studio with the R statistical package (Release 4.3.1) and module agricolae. Results were examined statistically using a one-way analysis of variance (ANOVA) by Tukey’s multiple range tests [38]. The differences between individual means were considered significant only if *p* < 0.05. Therefore, values in a figure followed by the same letter are not significantly different.

## 4. Discussion

The phytotoxic effect of essential oils has been widely reported in recent years [12,40,41,42,43,44,45,46,47,48,49] but, to our knowledge, few studies have focused on their mode(s) of action, in particular, for post-emergence treatment under greenhouse conditions [28,29]. It is important to keep in mind that while the fungicidal [50] and insecticidal [51] properties of cinnamon EO are well established, its phytotoxic effects on plants remain less explored. This study investigated its activity under greenhouse conditions against two indicator plants, namely a monocotyledon species, *Lolium perenne* L. (Poaceae), and a dicotyledon species, *Trifolium incarnatum* L. (Fabaceae). These plants can be considered part of the resident flora, weeds, or potential cover crop species. In line with our study, Bai et al., 2023 [37], found that among twelve commercial essential oils, garlic essential oil (GEO) sprayed on *Echinochloa crus-galli* (L.) had the most significant phytotoxic effect at variable concentration (0.01–0.1 g mL^−1^), which is more concentrated than our emulsion. This effect is explained by an inhibition of the antioxidant enzyme system and a reduced chlorophyll content.

Regarding the phytotoxic effect, leaves of *T. incarnatum* treated by cinnamon EO showed several chlorosis and necrosis after only 1 day. These symptoms are linked to the phytotoxic properties of the essential oils that cause contact injury and the formation of chlorophyllases, like observed by M’barek et al., 2019 [52]. Kaur et al., 2010 [53], confirmed that the necrosis is due to the loss of the cell membrane integrity of leaves.

To better understand these observed symptoms, the ultrastructure of leaf cells of different tissues was observed using transmission electron microscopy and then confirmed by biochemical tests. In fact, microscopic observations clearly showed that cinnamon EO caused damage to the plasma membrane structure, which became separated from the cell wall. These results were confirmed by the membrane integrity test, since cinnamon EO caused a significant electrolyte leakage compared to the control. That is what Dayan et al., 1999 [54], found when they evaluated the membrane integrity of cucumber treated by Dehydrozaluzanin C, a natural sesquiterpene lactone. As described in the literature, the plasma membrane plays an important role in protecting cells from the extracellular environment [55]. Most compounds from essential oils are small and can quickly reach the plasma membrane. For this reason, there is a gap in the literature about the interaction between molecules and the plasma membrane, so more studies are needed to understand essential oils’ mode of action. For example, molecular dynamic simulations have demonstrated that cinnamaldehyde, the lead compound in cinnamon EO, can specifically target the polar heads of model plasma membranes, potentially causing disruption. This penetration of the membrane surface allows cinnamaldehyde to interact with membrane receptors and ion channels [34]. In addition, lipid peroxidation and high leakage of electrolytes resulting in a loss of membrane integrity are among the key factors determining cellular injury [56].

Another effect observed in the leaf cells of *T. incarnatum* is the disappearance and collapse of the vacuole. These changes could be interpreted as cell plasmolysis induced by the desiccant effect of cinnamon EO. As expected, we found also that it significantly decreased the water content of the leaves of *T. incarnatum* and *L. perenne* compared to the control. The response of *T. incarnatum* to the desiccant effect of cinnamon EO was stronger and more severe than that of *L. perenne*. This could be explained by the difference in the chemical composition of the cuticle, cell wall, and plasma membrane between monocotyledons and dicotyledons [57,58]. Ben Kaab et al., 2020 [38], also found that *L. perenne* is more resistant than *T. incarnatum* in response to a crude methanolic extract of *Cynara cardunculus* leaves. In the same vein, Matsoukova et al., 2019 [59], confirmed that monocotyledonous species are more resistant to EOs than dicotyledonous species through the synthesis of osmolytes and ROS (reactive oxygen species) scavengers.

From the study of Synowiec et al., 2019 [60], it has been concluded that *Carum carvi* L. EO can function as desiccant bioherbicides, disrupting the leaf cuticular wax layer, leading to changes in leaf membrane integrity, dehydration, and, ultimately, cell death. After transmission electron microscopy observations, that is what our further physiological and biochemical analysis also showed. In fact, spraying cinnamon EO on weeds induced quick oxidative stress in treated leaves by increasing the MDA content in leaves and alterating the membrane integrity. Ben Ghnaya et al., 2013 [61], demonstrated that the active compounds contained in essential oils could cross cell membranes, interacting with membrane enzymes and proteins such as the H+-ATPase membrane pump, producing an outward flow of protons that would induce changes in the cells and, ultimately, their death. This is in agreement with previous reports showing that any change in membrane integrity could affect many biochemical functions and consequently result in the increase in oxidative stress parameters [43]. For example, the disturbance of the membrane causes potassium leakage that would inhibit glucose-dependent respiration. In addition, an alteration in water uptake regulated by proton pumps through the plasmalemmas of root cells would cause multiple physiological consequences such as slowed plant growth [62]. Thus, a high level of lipid peroxidation may be related to the inhibition of two membrane-associated enzymes, H+-ATPase and NADPH oxidase [63].

On the other hand, microscopic observations have clearly indicated damage to the chloroplasts, which play a crucial role in photosynthesis. Any alteration in its functions could disturb all biochemical and physiological processes in plants, in particular, those that depend on energy, such as cellular respiration [64]. This result was confirmed by the chlorophyll fluorescence measurement, since cinnamon EO at 6% completely reduced the fluorescence of *T. incarnatum* after only 15 min. Indeed, as stated by Soltys et al., 2013 [65], Ben Kaab et al., 2020 [38], and Verdeguer et al., 2020 [47], the presence of allelochemicals, especially essential oils, disrupts the photosynthesis process.

In contrast to the effect of cinnamon EO, glyphosate did not show an alteration of *T. incarnatum* metabolism, especially after 5 h. The desiccant effect was observed only after 3 days. It is well known that a glyphosate formulation is applied as a systemic herbicide and consequently takes time to react in the plant. Its mechanism involves targeting the 5-enolpyruvylshikimate-3-phosphate synthase (EPSPS) enzyme, thereby disrupting the shikimate pathway leading to cell death [66].

Regarding the potential alteration in plant metabolism in *T. incarnatum* caused by cinnamon EO, results have clearly showed an alteration in the plant metabolism of *T. incarnatum* and confirm again the disturbance of plant metabolism induced by the presence of cinnamaldehyde. This was confirmed by the disappearance in Raman spectra profile of some scattering bands which correspond to some primary metabolites such as carbohydrates and proteins. In addition, we also found two additional scattering bands attributed to molecules coming from the phenylalanine ammonia–lyase pathway and corresponding to phenylpropanoids compounds [67]. On the other hand, Rys et al., 2024 [29], studied the phytotoxic effect of nanoemulsions based on caraway EO, sourced from *Carum carvi* and using Fourier-transformed (FT) Raman spectroscopy. They found that this nanoemulsion at 1.5%, 2%, and 5% of EO revealed elevated levels of monosaccharides, originating from the hydrolysis of polysaccharides, within the endosperm of maize and barnyard grass seedlings.

Two main factors could potentially influence the effect of cinnamon EO. Firstly, the variation in the chemical composition of essential oils (EOs) can significantly affect their phytotoxic efficacy because the concentration of the lead compounds in EOs can differ substantially among different plant genotypes. For instance, the concentrations of the three major components, 1,8-cineole, camphor, and borneol, in *Rosmarinus officinalis* can vary from 26.0% to 51.2%, 4.9% to 29.7%, and 3.3% to 10%, respectively [68]. Ben Ghnaya et al., 2013 [61], explained this variability, concluding that the chemical composition of EO is influenced by several environmental factors, including climate, season, soil composition, genetic diversity of the species, geographical conditions, the harvest period, and the isolation technique of the EO. That is why it is necessary to carry out field efficiency tests. Secondly, the emulsifiers used to mix EO in water can also impact the phytotoxic effect of the EO. For example, Todero et al., 2018 [69], demonstrated that a formulation containing palm oil (sourced from *Elaeis guineensis*, *Arecaceae*), Tween 20, and Span 80 enhanced the phytotoxic effect of metabolites from *Phoma* sp. In agreement with this, Ben Kaab et al., 2019 [44], set up a herbicide formulation to enhance the phytotoxic effect of *Rosmarinus officinalis* (Lamiaceae) EO in post-emergence applications.

To summarize the mechanism of action of cinnamon EO on plant species, the observed water loss percentage and plasmolysis in cells confirm its desiccation effect. This is linked to the loss of membrane integrity, as cinnamaldehyde, a small lipophilic molecule, easily diffuses into cells and binds to membrane systems [34]. This disruption induces oxidative stress, confirmed by the high levels of MDA found in plant leaves, marked by the release of reactive oxygen species (ROS), leading to a “burn-down” effect, as detailed by Dayan et al. (2011) [70] and confirmed by Duke et al. (2024) [71].

## 5. Conclusions

Our results confirmed that cinnamon EO is a promising natural post-emergence bioherbicide. The phytotoxic effect of cinnamon EO was studied for the first time, which confirmed the rapid desiccant effect explained by cell plasmolysis, high oxidative stress, and extensive chloroplast and membrane damage. Raman analysis confirms the alteration of plant metabolism. Further studies will explore more this mechanism of action by studying the response of plants using low concentrations to identify the first differential proteins expressed in plants treated with cinnamon EO. Finally, this emulsion can be used in a new bioherbicide formulation (botanical) that could be integrated into integrated weed management (IWM) strategies. However, challenges remain, including the need to study its mechanism of action in plants at the proteomic and transcriptomic levels, as well as evaluate the environmental and ecotoxicological risks.

## Figures and Tables

**Table 1:** Vibrational bands and their assignments for spectra collected from *T. incarnatum* leaves, stems, and roots.

Band (cm^−1^) (Maximum Scattering Intensity)	Vibrational Mode	Main Assignment	References
522	ν(C–O–C) glycosidic	Cellulose	[35]
1000	In-plane CH_3_ rocking of polyene aromatic ring of phenylalanine	Carbohydrates, proteins	[35]
1048	ν(C–O) + ν(C–C) + δ(C–O–H)	Cellulose, phenylpropanoids	[35]
1096	ν(C–O) + ν(C–C) + δ(C–O–H)	Carbohydrates	[29]
1155	C–C stretching; v(C–O–C), v(C–C) in glycosidic linkages, asymmetric ring breathing	Carbohydrates, carotenoids	[35]
1440	δ(CH_2_) + δ(CH_3_)	Phenylpropanoids	[35]
1521	ν1 C=C	Carotenoides	[35,36]
1606	(C–C) aromatic ring + σ(CH)	Phenylpropanoids	[35]
1653	Ν_conj_ C=O	coniferylaldehyde, lignin	[36]
2859, 2930	C–H stretching	Glucides, carotenoids, aliphatics Phenylpropanoids, proteins	[35]

Abbreviations: ν: stretching vibration; δ: deformation vibration; Conj: conjugated.

## Data Availability

The data presented in this study are available on request from the corresponding author.

## References

[1] van Evert F.K., Fountas S., Jakovetic D., Crnojevic V., Travlos I., Kempenaar C. (2017). Big Data for Weed Control and Crop Protection. Weed Res..

[2] Watanabe Y., Novaes P., Varela R.M., Molinillo J.M.G., Kato-Noguchi H., Macias F.A. (2014). Phytotoxic Potential of *Onopordum acanthium* L. (Asteraceae). Chem. Biodivers..

[3] Idziak R., Waligóra H., Szuba V. (2022). The Influence of Agronomical and Chemical Weed Control on Weeds of Corn. J. Plant Prot. Res..

[4] Ganie Z.A., Soltani N., Mckenzie-Gopsill A.G., Felix J., Hutchinson P.J.S., Dille J.A.J., Sikkema P.H. (2023). Potential Potato Yield Loss from Weed Interference in the United States and Canada. Weed Technol..

[5] Umer M., Khan N.M., Razaq Z., Nissa M.U., Anwar N., Ali M.A., Bhatti A.M., Khan M., Abbas A. (2022). Bio-Herbicides: Development, Use and Elucidation of the Factors Affecting Their Efficacy. Plant Prot..

[6] Ogunmoyole T., Adeyeye R.I., Olatilu B.O., Akande O.A., Agunbiade O.J. (2019). Multiple Organ Toxicity of Datura Stramonium Seed Extracts. Toxicol. Rep..

[7] Assadpour E., Can Karaça A., Fasamanesh M., Mahdavi S.A., Shariat-Alavi M., Feng J., Kharazmi M.S., Rehman A., Jafari S.M. (2023). Application of Essential Oils as Natural Biopesticides; Recent Advances. Crit. Rev. Food Sci. Nutr..

[8] Scavo A., Rial C., Varela R.M., Molinillo J.M.G., Mauromicale G., Macias F.A. (2019). Influence of Genotype and Harvest Time on the *Cynara cardunculus* L. Sesquiterpene Lactone Profile. J. Agric. Food Chem..

[9] Alipour M., Saharkhiz M.J. (2016). Phytotoxic Activity and Variation in Essential Oil Content and Composition of Rosemary (*Rosmarinus officinalis* L.) during Different Phenological Growth Stages. Biocatal. Agric. Biotechnol..

[10] Zhu X., Han C., Gao T., Shao H. (2016). Chemical Composition, Phytotoxic and Antimicrobial Activities of the Essential Oil of *Scutellaria strigillosa* Hemsley. J. Essent. Oil-Bear. Plants.

[11] Bozhuyuk A.U. (2020). Herbicidal Activity and Chemical Composition of Two Essential Oils on Seed Germinations and Seedling Growths of Three Weed Species. J. Essent. Oil-Bear. Plants.

[12] Jiang C., Zhou S., Liu L., Toshmatov Z., Huang L., Shi K., Zhang C., Shao H. (2021). Evaluation of the Phytotoxic Effect of the Essential Oil from Artemisia Absinthium. Ecotoxicol. Environ. Saf..

[13] Somala N., Laosinwattana C., Teerarak M. (2022). Formulation Process, Physical Stability and Herbicidal Activities of Cymbopogon Nardus Essential Oil-Based Nanoemulsion. Sci. Rep..

[14] Li J., Chen H., Guo C., Chen Q., Zhao T., Chen X., Du Y., Du H., Miao Y., Liu D. (2023). Artemisia Argyi Essential Oil Exerts Herbicidal Activity by Inhibiting Photosynthesis and Causing Oxidative Damage. Ind. Crops Prod..

[15] Cordeau S., Triolet M., Wayman S., Steinberg C., Guillemin J.P. (2016). Bioherbicides: Dead in the Water? A Review of the Existing Products for Integrated Weed Management. Crop Prot..

[16] Pavela R., Benelli G. (2016). Essential Oils as Ecofriendly Biopesticides? Challenges and Constraints. Trends Plant Sci..

[17] Bakkali F., Averbeck S., Averbeck D., Idaomar M. (2008). Biological Effects of Essential Oils—A Review. Food Chem. Toxicol..

[18] Heleno S.A., Martins A., João M., Queiroz R.P., Ferreira I.C.F.R. (2015). Bioactivity of Phenolic Acids: Metabolites versus Parent Compounds: A Review. FOOD Chem..

[19] Sharifi-Rad J., Dey A., Koirala N., Shaheen S., El Omari N., Salehi B., Goloshvili T., Cirone Silva N.C., Bouyahya A., Vitalini S. (2021). Cinnamomum Species: Bridging Phytochemistry Knowledge, Pharmacological Properties and Toxicological Safety for Health Benefits. Front. Pharmacol..

[20] de Oliveira M.M.M., Brugnera D.F., do Nascimento J.A., Batista N.N., Piccoli R.H. (2012). Cinnamon Essential Oil and Cinnamaldehyde in the Control of Bacterial Biofilms Formed on Stainless Steel Surfaces. Eur. Food Res. Technol..

[21] Chou S.T., Chang W.L., Chang C.T., Hsu S.L., Lin Y.C., Shih Y. (2013). Cinnamomum Cassia Essential Oil Inhibits α-MSH-Induced Melanin Production and Oxidative Stress in Murine B16 Melanoma Cells. Int. J. Mol. Sci..

[22] Wu S.J., Ng L.T. (2011). Antiproliferative Activity of Cinnamomum Cassia Constituents and Effects of Pifithrin-Alpha on Their Apoptotic Signaling Pathways in Hep G2 Cells. Evid.-Based Complement. Altern. Med..

[23] Pannee C., Chandhanee I., Wacharee L. (2014). Antiinflammatory Effects of Essential Oil from the Leaves of Cinnamomum Cassia and Cinnamaldehyde on Lipopolysaccharide-Stimulated J774A.1 Cells. J. Adv. Pharm. Technol. Res..

[24] Choi J., Lee K.T., Ka H., Jung W.T., Jung H.J., Park H.J. (2001). Constituents of the Essential Oil of the Cinnamomum Cassia Stem Bark and the Biological Properties. Arch. Pharm. Res..

[25] De Clerck C., Maso S.D., Parisi O., Dresen F., Zhiri A., Haissam Jijakli M. (2020). Screening of Antifungal and Antibacterial Activity of 90 Commercial Essential Oils against 10 Pathogens of Agronomical Importance. Foods.

[26] Lee J.E., Jung M., Lee S.C., Huh M.J., Seo S.M., Park I.K. (2020). Antibacterial Mode of Action of Trans-Cinnamaldehyde Derived from Cinnamon Bark (*Cinnamomum verum*) Essential Oil against Agrobacterium Tumefaciens. Pestic. Biochem. Physiol..

[27] Araniti F., Lo D., Graña E., Teijeira M., Rey M., Gonza V., Reigosa M.J., Sa A.M. (2023). Trans-Cinnamaldehyde-Related Overproduction of Benzoic Acid and Oxidative Stress on *Arabidopsis thaliana*. Front. Plant Sci..

[28] Verdeguer M., Sánchez-Moreiras A.M., Araniti F. (2020). Phytotoxic Effects and Mechanism of Action of Essential Oils and Terpenoids. Plants.

[29] Rys M., Miastkowska M., Lorenzo P., Synowiec A., Łętocha A., Bonikowska A.W. (2024). The Effect of Caraway Oil—Loaded Bio—Nanoemulsions on the Growth and Performance of Barnyard Grass and Maize. Sci. Rep..

[30] Baena J.R., Lendl B. (2004). Raman Spectroscopy in Chemical Bioanalysis. Curr. Opin. Chem. Biol..

[31] Fernández Pierna J.A., Vermeulen P., Amand O., Tossens A., Dardenne P., Baeten V. (2012). NIR Hyperspectral Imaging Spectroscopy and Chemometrics for the Detection of Undesirable Substances in Food and Feed. Chemom. Intell. Lab. Syst..

[32] Tworkoski T. (2002). Herbicide Effects of Essential Oils. Weed Sci..

[33] Werrie P.Y., Juillard A., Heintz C., Brisset M.N., Fauconnier M.L. (2022). Phytotoxicity and Plant Defence Induction by Cinnamomum Cassia Essential Oils Application on Malus Domestica Tree: A Molecular Approach. Agronomy.

[34] Lins L., Dal Maso S., Foncoux B., Kamili A., Laurin Y., Genva M., Jijakli M.H., De Clerck C., Fauconnier M.L., Deleu M. (2019). Insights into the Relationships Between Herbicide Activities, Molecular Structure and Membrane Interaction of Cinnamon and Citronella Essential Oils Components. Int. J. Mol. Sci..

[35] Payne W.Z., Kurouski D. (2021). Raman Spectroscopy Enables Phenotyping and Assessment of Nutrition Values of Plants: A Review. Plant Methods.

[36] Zeise I., Heiner Z., Holz S., Joester M., Büttner C., Kneipp J. (2018). Raman Imaging of Plant Cell Walls in Sections of Cucumis Sativus. Plants.

[37] Bai H., Ni X., Han J., Luo D., Hu Y., Jin C., Li Z. (2023). Phytochemical Profiling and Allelopathic Effect of Garlic Essential Oil on Barnyard Grass (*Echinochloa crusgalli* L.). PLoS ONE.

[38] Ben Kaab S., Lins L., Hanafi M., Rebey I.B., Deleu M., Fauconnier M.-L., Ksouri R., Jijakli M.H., De Clerck C. (2020). Cynara Cardunculus Crude Extract as a Powerful Natural Herbicide and Insight into the Mode of Action of Its Bioactive Molecules. Biomolecules.

[39] Poonpaiboonpipat T., Pangnakorn U., Suvunnamek U., Teerarak M., Charoenying P., Laosinwattana C. (2013). Phytotoxic Effects of Essential Oil from Cymbopogon Citratus and Its Physiological Mechanisms on Barnyardgrass (*Echinochloa crus-galli*). Ind. Crops Prod..

[40] De Almeida L.F.R., Frei F., Mancini E., De Martino L., De Feo V. (2010). Phytotoxic Activities of Mediterranean Essential Oils. Molecules.

[41] Casella F., Vurro M., Valerio F., Perrino E.V., Mezzapesa G.N., Boari A. (2023). Phytotoxic Effects of Essential Oils from Six Lamiaceae Species. Agronomy.

[42] Kashkooli A.B., Saharkhiz M.J. (2014). Essential Oil Compositions and Natural Herbicide Activity of Four Denaei Thyme (*Thymus daenensis* Celak.) Ecotypes. J. Essent. Oil Bear. Plants.

[43] Hazrati H., Saharkhiz M.J., Niakousari M., Moein M. (2017). Natural Herbicide Activity of *Satureja hortensis* L. Essential Oil Nanoemulsion on the Seed Germination and Morphophysiological Features of Two Important Weed Species. Ecotoxicol. Environ. Saf..

[44] Ben Kaab S., Rebey I.B., Hanafi M., Berhal C., Fauconnier M.L., De Clerck C., Ksouri R., Jijakli H. (2019). *Rosmarinus officinalis* Essential Oil as an Effective Antifungal and Herbicidal Agent. Spanish J. Agric. Res..

[45] Silva E.R., Igartuburu J.M., Overbeck G.E., Soares G.L.G., Macías F.A. (2020). Bioherbicide Potential of *Eucalyptus saligna* Leaf Litter Essential Oil. Chem. Biodivers..

[46] Taban A., Saharkhiz M.J., Khorram M. (2020). Formulation and Assessment of Nano-Encapsulated Bioherbicides Based on Biopolymers and Essential Oil. Ind. Crops Prod..

[47] Verdeguer M., Torres-Pagan N., Muñoz M., Jouini A., García-Plasencia S., Chinchilla P., Berbegal M., Salamone A., Agnello S., Carrubba A. (2020). Herbicidal Activity of *Thymbra capitata* (L.) Cav. Essential Oil. Molecules.

[48] Abd-Elgawad A.M., El Gendy A.E.N.G., Assaeed A.M., Al-Rowaily S.L., Alharthi A.S., Mohamed T.A., Nassar M.I., Dewir Y.H., Elshamy A.I. (2021). Phytotoxic Effects of Plant Essential Oils: A Systematic Review and Structure-Activity Relationship Based on Chemometric Analyses. Plants.

[49] Kordali S., Kabaagac G., Sen İ., Yilmaz F., Najda A. (2022). Phytotoxic Effects of Three Origanum Species Extracts and Essential Oil on Seed Germinations and Seedling Growths of Four Weed Species. Agronomy.

[50] Wang Y., Zhao R., Yu L., Zhang Y., He Y., Yao J. (2014). Evaluation of Cinnamon Essential Oil Microemulsion and Its Vapor Phase for Controlling Postharvest Gray Mold of Pears (*Pyrus pyrifolia*). J. Sci. Food Agric..

[51] Viteri Jumbo L.O., Faroni L.R.A., Oliveira E.E., Pimentel M.A., Silva G.N. (2014). Potential Use of Clove and Cinnamon Essential Oils to Control the Bean Weevil, Acanthoscelides Obtectus Say, in Small Storage Units. Ind. Crops Prod..

[52] M’barek K., Zribi I., Ullah M.J., Haouala R. (2019). The Mode of Action of Allelochemicals Aqueous Leaf Extracts of Some Cupressaceae Species on Lettuce. Sci. Hortic..

[53] Kaur S., Singh H.P., Mittal S., Batish D.R., Kohli R.K. (2010). Phytotoxic Effects of Volatile Oil from Artemisia Scoparia against Weeds and Its Possible Use as a Bioherbicide. Ind. Crops Prod..

[54] Dayan F.E., Tellez M.R., Galindo J.C.G., Herna A., Macõâ F.A., Paul R.N., Duke S.O. (1999). Dehydrozaluzanin C, a Natural Sesquiterpenolide, Causes Rapid Plasma Membrane Leakage. Phytochemistry.

[55] Mani-López E., Cortés-Zavaleta O., López-Malo A. (2021). A Review of the Methods Used to Determine the Target Site or the Mechanism of Action of Essential Oils and Their Components against Fungi. SN Appl. Sci..

[56] Batish D.R., Singh H.P., Setia N., Kaur S., Kohli R.K. (2006). 2-Benzoxazolinone (BOA) Induced Oxidative Stress, Lipid Peroxidation and Changes in Some Antioxidant Enzyme Activities in Mung Bean (*Phaseolus aureus*). Plant Physiol. Biochem..

[57] Masuda Y. (1985). Cell Wall Modifications during Auxin-Induced Cell Extension in Monocotyledonous and Dicotyledonous Plants. Biol. Plant..

[58] Carpita N.C. (1996). Structure and Biogenesis of the Cell Walls of Grasses. Annu. Rev. Plant Physiol. Plant Mol. Biol..

[59] Matoušková M., Jurová J., Grul’ová D., Wajs-Bonikowska A., Renčo M., Sedlák V., Poráčová J., Gogal’ová Z., Kalemba D. (2019). Phytotoxic Effect of Invasive *Heracleum mantegazzianum* Essential Oil on Dicot and Monocot Species. Molecules.

[60] Synowiec A., Możdżeń K., Krajewska A., Landi M., Araniti F. (2019). Carum Carvi L. Essential Oil: A Promising Candidate for Botanical Herbicide against *Echinochloa crus-galli* (L.) P. Beauv. in Maize Cultivation. Ind. Crops Prod..

[61] Ben Ghnaya A., Hanana M., Amri I., Balti H., Gargouri S., Jamoussi B., Hamrouni L. (2013). Chemical Composition of *Eucalyptus erythrocorys* Essential Oils and Evaluation of Their Herbicidal and Antifungal Activities. J. Pest Sci..

[62] Hejl A.M., Koster K.L. (2004). The Allelochemical Sorgoleone Inhibits Root H+-ATPase and Water Uptake. J. Chem. Ecol..

[63] Cruz-Ortega R., Lara-Núñez A., Anaya A.L. (2007). Allelochemical Stress Can Trigger Oxidative Damage in Receptor Plants. Plant Signal. Behav..

[64] Araniti F., Miras-Moreno B., Lucini L., Landi M., Abenavoli M.R. (2020). Metabolomic, Proteomic and Physiological Insights into the Potential Mode of Action of Thymol, a Phytotoxic Natural Monoterpenoid Phenol: The Phytotoxic Effect of Thymol on Adult Plants of A. Thaliana. Plant Physiol. Biochem..

[65] Soltys D., Krasuska U., Bogatek R., Gniazdowska A. (2013). Allelochemicals as Bioherbicides—Present and Perspectives. Herbic.-Curr. Res. Case Stud. Use.

[66] Gomes M.P., Smedbol E., Chalifour A., Hénault-Ethier L., Labrecque M., Lepage L., Lucotte M., Juneau P. (2014). Alteration of Plant Physiology by Glyphosate and Its By-Product Aminomethylphosphonic Acid: An Overview. J. Exp. Bot..

[67] Zaio Y.P., Gatti G., Ponce A.A., Saavedra Larralde N.A., Martinez M.J., Zunino M.P., Zygadlo J.A. (2018). Cinnamaldehyde and Related Phenylpropanoids, Natural Repellents, and Insecticides against Sitophilus Zeamais (Motsch.). A Chemical Structure-Bioactivity Relationship. J. Sci. Food Agric..

[68] Zaouali Y., Bouzaine T., Boussaid M. (2010). Essential Oils Composition in Two *Rosmarinus officinalis* L. Varieties and Incidence for Antimicrobial and Antioxidant Activities. Food Chem. Toxicol..

[69] Todero I., Confortin T.C., Luft L., Brun T., Ugalde G.A., de Almeida T.C., Arnemann J.A., Zabot G.L., Mazutti M.A. (2018). Formulation of a Bioherbicide with Metabolites from *Phoma* sp. Sci. Hortic..

[70] Dayan F.E., Owens D.K., Duke S.O. (2012). Rationale for a Natural Products Approach to Herbicide Discovery. Pest Manag. Sci..

[71] Duke S.O., Twitty A., Baker C., Sands D., Boddy L., Travaini M.L., Sosa G., Polidore A.L.A., Jhala A.J., Kloeber J.M. (2024). New Approaches to Herbicide and Bioherbicide Discovery. Weed Sci..

